# Advances in Ozone-Based Inactivation of SARS-CoV-2: An Updated Review

**DOI:** 10.3390/ijms27083632

**Published:** 2026-04-18

**Authors:** Karyne Rangel, Maria Helena Simões Villas-Bôas, Salvatore Giovanni De-Simone

**Affiliations:** 1 Center for Technological Development in Health (CDTS), National Institute of Science and Technology for Innovation in Neglected Population Diseases (INCT-IDPN), Oswaldo Cruz Foundation (FIOCRUZ), Rio de Janeiro 21040-900, RJ, Brazil; 2 Laboratory of Epidemiology and Molecular Systematics (LESM), Oswaldo Cruz Institute, Oswaldo Cruz Foundation (FIOCRUZ), Rio de Janeiro 21040-900, RJ, Brazil; 3 Microbiology Department, National Institute for Quality Control in Health (INCQS), Oswaldo Cruz Foundation (FIOCRUZ), Rio de Janeiro 21040-900, RJ, Brazil; maria.villas@fiocruz.br; 4 Post-Graduation Program in Science and Biotechnology, Department of Molecular and Cellular Biology, Biology Institute, Federal Fluminense University (U.F.F.), Niteroi 22040-036, RJ, Brazil

**Keywords:** ozone, gaseous, aqueous, COVID-19, SARS-CoV-2, disinfection

## Abstract

The onset of the COVID-19 pandemic prompted the rapid development and deployment of novel strategies and methodologies to manage the dissemination of microorganisms. Understanding the crucial role that contaminated surfaces play in the spread of viruses highlights the importance of having effective cleaning and disinfection protocols in place for inanimate objects. A variety of antimicrobial agents have shown strong effectiveness against the SARS-CoV-2 virus. Various factors can impact on the performance of these agents. As a result, technologies utilizing ozone’s microbicidal effects have been developed or improved for cleaning indoor areas, surfaces, and materials, despite ozone’s diverse uses being known for years. Ozone offers the advantage of adaptability for both gaseous and aqueous use, depending on the nature of the decontaminated surfaces. Moreover, ozone-infused water is ecologically benign, possesses microbial-fighting capabilities, and synergistically reinforces the biocidal action of other chemical disinfectants. This review aims to summarize the efforts dedicated to harnessing gaseous and aqueous ozone as a valuable means to eliminate the SARS-CoV-2 virus from environments, surfaces, clinical equipment, and office supplies. This review sourced evidence-based articles from electronic databases, including MEDLINE (via PubMed), EMBASE, the Cochrane Library (CENTRAL), and preprint repositories. The findings illustrated that ozone could serve as an additional tool for curbing the proliferation of COVID-19 and other viral infections. Additionally, we elucidated the operational attributes of ozone, the variables that influence its disinfection potency, and the mechanisms of its virucidal action. Notably, this review does not encompass the disinfection of the COVID-19 virus in wastewater.

## 1. Introduction

The ongoing global health crisis caused by the Severe Acute Respiratory Syndrome Coronavirus 2 (SARS-CoV-2) and the resulting COVID-19 pandemic has profoundly impacted societies and economies worldwide, leading to millions of deaths [1]. While quantitative risk-assessment studies conclude that the probability of infection from contaminated surfaces, such as fomite transmission, is relatively minimal compared to direct person-to-person airborne transmission, concerns about microbial contamination have risen significantly during this pandemic. This heightened concern stems from the operational significance of surface transmission; even a low-probability risk becomes a significant public health concern when dealing with a highly infectious virus on a global scale. Since the onset of the pandemic, the emergence of new variants has continuously posed challenges for prevention and control efforts. Alongside the primary route of direct transmission, the potential risk of indirect transmission through contaminated surfaces and objects warrants attention [2,3,4,5]. These surfaces may indeed contribute to the spread of the virus, particularly in healthcare settings or high-touch public areas, justifying its consideration in mitigation strategies. It is important to note that this review focuses on the disinfection of air, surfaces, and fomites; the specific application of ozone for wastewater disinfection is beyond its scope [6,7,8].

Beyond direct person-to-person contact, research indicates that SARS-CoV-2 can spread through liquid droplets (short-range) and aerosols (long-range) when an infected individual talks, sings, breathes heavily, coughs, or sneezes [9]. Aerosol particles containing the virus can remain suspended indoors in closed or poorly ventilated spaces for at least 3 h [8]. Meanwhile, larger droplets, known as flügge droplets, do not disperse beyond 1.5 to 2 m from the source and remain airborne for a limited duration [7]. These emitted particles can settle on environmental surfaces and remain viable for hours to days, potentially leading to indirect transmission when individuals touch these surfaces and subsequently come into contact with their eyes, nose, or mouth [5,8]. SARS-CoV-2 can persist on hospital and household surfaces for up to 7 days [7]. Experimental studies have also demonstrated the virus’s ability to survive on various materials such as plastic, latex, glass, and metal for hours to days [10].

The advent of the COVID-19 pandemic underscored the importance of environmental-microbiological prevention and monitoring systems in responding to health crises and other threats [11,12,13]. While the initial vaccines have proven effective in reducing hospitalizations, infections, and deaths associated with COVID-19 [14,15,16], the constant emergence of new viral variants poses ongoing challenges [17,18]. Therefore, developing methods to ensure the disinfection of air, surfaces, fomites, and medical equipment has become paramount in interrupting the transmission chain. Implementing effective disinfection strategies remains crucial to controlling local transmissions through human-to-human contact, contaminated surfaces, or airborne particles. New disinfection approaches have the potential to significantly decrease case numbers during the current pandemic and future outbreaks.

The demand for virucidal disinfectants has surged since the outbreak of COVID-19, with more than five hundred disinfectants approved by the Environmental Protection Agency proving effective against SARS-CoV-2. However, their widespread use has raised concerns due to negative impacts on ecosystems and human health [19]. In this context, ozone disinfection is a viable option, having demonstrated high efficacy in eliminating bacteria, fungi, molds, and viruses, including the SARS virus [20,21,22,23]. It is important to note that prolonged exposure to high ozone concentrations can harm the human respiratory tract and damage construction materials, impacting air quality [24,25]. In Italy, the Ministry of Health has recognized ozone as a natural aid for sanitizing contaminated environments, further reinforcing its social legitimacy through the protocol issued on 31 July 1996 (24,482) [26].

It is essential to differentiate between the effects of gaseous ozone used for environmental disinfection and its activity within the human body [27,28]. While the primary aim of this review is to discuss environmental disinfection, it is pertinent to acknowledge that the pandemic has also shed light on the possible advantages of ozone therapy for treating COVID-19 patients. This approach has shown encouraging results, including helping to protect the heart and kidneys, improving oxygen flow in the body, boosting the immune system, reducing the risk of blood clots, and slowing down the virus’s ability to spread [29,30]. Recent studies suggest that ozone could be an alternative or adjuvant medical treatment for COVID-19 patients when used in conjunction with standard anti-inflammatory medications [31,32,33,34]. Ozone therapy involves creating a standardized mixture of oxygen and ozone (O2/O3), known as medicinal ozone [35]. The effects of ozone are primarily mediated through antioxidant systems, resulting in significant anti-inflammatory, immunomodulatory, and antiviral properties, as well as direct effects on coagulation and enhancements in microcirculation [36,37]. In the medical field, ozonated water is utilized for disinfecting surfaces and equipment, providing substantial advantages in dentistry through its efficacy in inactivating viruses, including coronaviruses [38,39,40,41,42]. The action of ozone is primarily dependent on direct contact with pathogens, with a radical mechanism becoming increasingly pronounced at pH levels exceeding 7 [43].

This mechanism holds particular importance for the localized treatment of infected wounds, as it facilitates the effective elimination of microorganisms and promotes wound cleansing, both of which are essential for the healing process [44]. In light of these considerations, the current review aims to elucidate the efficacy of ozone gas in inactivating or diminishing SARS-CoV-2 viruses on various surfaces, fomites, and in the air, while also assessing its potential to mitigate cross-contamination. Furthermore, the review will discuss the operational characteristics of ozone, the factors that influence its disinfection capabilities, and the mechanisms that underlie its virucidal actions.

## 2. Ozone Characteristics

Ozone (O3), a naturally occurring arrangement of three oxygen atoms (a triatomic gas and an oxygen allotrope), is a potent and environmentally friendly oxidizing agent known for its distinct pungent odor. It has a half-life of approximately one hour at room temperature and breaks down spontaneously to produce oxygen [45]. While ozone gas is bluish at room temperature, its presence is often not visually apparent. At a chilling −112 °C, condensed ozone transforms into a potentially explosive, dark-blue liquid [46]. Notably, ozone is significantly less stable than atmospheric oxygen [28], which means it does not accumulate and must be generated on demand through ozone generation systems [47,48]. Various techniques are used for ozone production, including Arc Discharge, dielectric barrier discharge, ultraviolet lamps, and electrostatic discharge/filter methods [49,50,51,52,53,54].

Ozone swiftly decomposes into oxygen in both water and air. It boasts a higher oxidative potential (2.07 V) than other oxidants such as potassium permanganate and chlorine. This high oxidative potential enables ozone to effectively oxidize a wide range of organic and inorganic compounds [55]. Although ozone is only slightly soluble in water, it has a much higher solubility compared to oxygen. At standard pressure and temperature, pure ozone dissolves at a remarkable rate of 641 mg/L, which is roughly 13 times more than oxygen [56]. In aquatic environments, ozone can produce reactive oxygen radicals through indirect oxidation processes [57]. This unique oxidative strength and rapid degradation make ozone a highly effective microbicidal agent, successfully targeting a broad spectrum of microorganisms, including bacteria, viruses, fungi, and protozoa [23,57,58,59,60].

As a result, ozone has a wide range of applications for disinfecting indoor spaces, surfaces, materials, food, and water. It can be used either in its gaseous form or dissolved in a solution, depending on the surface being targeted. Whether in aqueous or gaseous state, ozone is effective in decontaminating various surfaces. Hospital rooms can be disinfected using ozone gas, while dissolved ozone is used in the treatment of food and water [61]. Ozone’s microbicidal kinetics are faster than those of other oxidative agents used in chemical disinfectants, making it a safer alternative to chlorine, which generates harmful disinfection by-products and reacts significantly faster with organic matter [62]. Ozone has diverse applications across various industries due to its deodorizing, bleaching, and decontaminating properties.

## 3. Factors Influencing Ozone Disinfection

Ozone is a powerful substance that can kill many different germs and harmful microorganisms. However, the way it affects each type of germ can vary, depending on its unique biological makeup. The antimicrobial performance of ozone can be influenced by several parameters (as outlined in Table 1), including temperature, pressure, relative humidity, pH, conductivity, and the composition of organic matter [61]. In the context of surface disinfection, factors like ambient humidity, response time, material types, microorganism characteristics, ambient temperature, and surface properties all impact the efficacy of ozone disinfection [57]. The environmental factors of temperature, pressure, and relative humidity have a significant impact on gaseous ozonation. Additionally, factors such as pH, conductivity, and the composition of organic matter can significantly affect the effectiveness of aqueous ozone. Gaseous ozone is more effective at inactivating microorganisms when relative humidity is high [4,63].

Furthermore, the chemical composition, texture, and shape of the treated surface are crucial considerations for ozone disinfection. Material properties, whether absorbent or non-absorbent, the method of contamination (wet, dry, or droplets), the ozone generation technique, the type of microorganism, and the exposure dosage (ozone concentration over exposure duration) are key factors directly impacting the efficiency of the ozonation process [60]. In terms of water disinfection, parameters such as microorganism characteristics, water temperature, response time, organic matter, pH value, and turbidity all require evaluation [64,65]. In the case of aerosol disinfection, the principal factors include the concentration–time (CT) value (measured in mg/L/min), droplet size, and ambient humidity [50]. The CT value serves as a key indicator for assessing the effectiveness of ozone disinfection, as it reflects the combined effect of ozone concentration over time. A lower CT value indicates greater disinfection strength, requiring less time to reach a set sterilization rate [61].

## 4. Ozone Virucidal Mechanism

Viruses, being compact and simple in structure, lack cellular components and typically contain just one type of nucleic acid (DNA or RNA). These obligatory parasites rely on host cells for replication. The replication cycle of coronaviruses begins with the adsorption phase, during which the receptor-binding domain (RBD) of the S1 subunit of the spike glycoprotein interacts with specific receptors on the host cell membrane. This receptor specificity varies among different coronavirus species. For example, SARS-CoV-2 targets the human angiotensin-converting enzyme 2 (ACE2) receptor, facilitating the fusion of the viral envelope with the host cell membrane and subsequently releasing the viral RNA genome into the cytoplasm [66,67].

**Figure 1:**
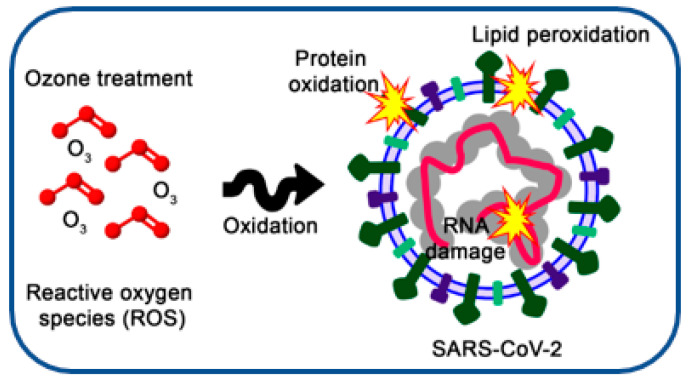
Potent mechanism of ozone’s virucidal action on SARS-CoV-2 virus via direct and indirect oxidation.

Ozone’s inactivation of viruses occurs through oxidative reactions with the fundamental biomolecules in these target organisms. This oxidation renders them incapable of initiating infections [68]. The virucidal potential of ozone also extends to aqueous environments [69,70]. Ozone’s impact encompasses damage to the viral envelope and genetic material. This process is accomplished through the direct oxidation of various molecules, peroxidation of phospholipids, and the generation of reactive oxygen species (ROS) [61]. Ozone and its ROS can oxidize various parts of enveloped or non-enveloped viruses, contributing to their inactivation (Figure 1). Like bacteria and fungi, ozone disrupts viruses through the peroxidation of lipids and proteins. This harms the viral lipid envelope and protein capsid, effectively neutralizing their ability to infect new cells [58]. A comprehensive review by Bayarri and colleagues highlights how ozone-mediated inactivation affects the molecular organization of viral genomes and the protein capsid [57]. The susceptibility of enveloped coronaviruses to ozone-induced damage might be greater due to the interaction between ozone and the lipid layers of their envelopes [71]. Ozone’s interaction with viral genomes involves molecular diffusion through external structures to reach the nucleic material. These external structures encompass the capsid and, in some cases, viral envelope proteins and lipids, as seen in the case of SARS-CoV-2 [72].

However, it is noteworthy that viruses are more resilient to ozone treatments than other microorganisms [73]. Molecular simulation studies have confirmed ozone’s ability to disrupt virus protein and lipid structures by targeting specific amino acids, such as cysteine, methionine, and tryptophan [51]. It is important to recognize that while knowledge of viral components contributes to understanding viral inactivation, it is not solely reliant on composition. Factors like viral structure and molecular organization play a role in determining the extent to which oxidants can access potential viral targets [74].

## 5. Inactivation of SARS-CoV-2 by Gaseous Ozone in Environments and on Surfaces

In response to the ongoing global COVID-19 pandemic, a comprehensive array of preventive and protective strategies has been implemented. Building on the success of ozone treatment against SARS-CoV-1 in 2002, researchers began investigating its effectiveness against SARS-CoV-2 due to the virus’ significant genetic similarity to SARS-CoV-1, at 80% [64]. As a result, ozone-based sanitation, in both gaseous and aqueous forms, has become a critical area of research, exploring its application in various environments, on surfaces, and on objects. This section focuses on studies examining ozone’s ability to neutralize the novel SARS-CoV-2 virus. It is essential to note that most of these studies utilized biologically safe viral surrogates that resemble SARS-CoV-2 in terms of form, function, and structure, as overseeing the live virus requires biosafety level 3 facilities [75,76].

Ozone gas has the potential to inactivate viruses present in contaminated spaces. A study found that ozone, high temperatures, and low humidity negatively affect coronavirus survival [77]. As ambient ozone concentration levels increased (from 48.83 to 94.67 μg/m^3^), together with relative humidity (23.33% to 82.67%) and temperature (−13.17 to 19 °C), a decrease in SARS-CoV-2 transmission was observed. Despite the low ozone concentration in the atmosphere lacking a direct inactivation effect, it displayed a relative inhibitory influence on the airborne transmission of SARS-CoV-2, thereby verifying the theoretical potential of utilizing active air disinfection to impede its spread [76]. De Forni et al. [78] used the ICON3 disinfection device to evaluate the effect of low ozone concentrations on inactivating SARS-CoV-2 isolates in an indoor environment devoid of individuals or animals. Findings indicated that exposure to low-concentration ozone (averaging 3.18 ppm) for 20 min could achieve over 99% inactivation of SARS-CoV-2. The study also subjected SARS-CoV-2 droplets (10, 3, and 0.5 μL) to varying ozone concentrations (ranging from 5.44 to 1.47 ppm) for 20 min, yielding significant inactivation results. A parallel assessment showed that ICON3’s ozone disinfection efficiency increases linearly with room volume in controlled rooms of 15, 30, and 60 m^3^. These findings suggested the potential utilization of ICON3 for SARS-CoV-2 disinfection in unoccupied indoor environments under controlled and secure conditions. A study evaluated the inactivating effects of hypochlorous acid, chlorine dioxide, and ozone on SARS-CoV-2 and influenza A virus in the air using a standardized assessment system. The results showed that viral infectivity titers decreased in a concentration- and time-dependent manner. Ozone at 1.0 ppm achieved an approximately 2 log reduction in SARS-CoV-2 infectivity within 10 min. However, SARS-CoV-2 remained detectable in the air under conditions where the influenza A virus was inactivated below detection limits. These findings highlight ozone’s effectiveness in reducing airborne SARS-CoV-2 and influenza A virus [79]. Albert et al. [27] quantified the efficacy, safety, and effectiveness of the aqueous ozone spray using a crewless aerial vehicle. Optimized flight parameters ensured coverage exceeding 97% on external surfaces. In spray operations employing 1 mg/L aqueous ozone, atmospheric ozone concentrations remained consistent with background levels (≤0.04 ppm). The study demonstrated the efficient inactivation of two SARS-CoV-2 strains in just 5 min under a 0.75 mg/L ozone concentration, while a 0.375 mg/L concentration achieved 82–91.5% inactivation. Results confirmed the capacity of aqueous ozone to effectively eliminate the virus while maintaining safety standards for both humans and the environment [27]. A recent investigation, which applied ozone treatment at varying concentrations to office supplies (e.g., personal computer monitors, keyboards, and computer mice) and clinical equipment (such as continuous positive airway pressure tubes and personal protective equipment), revealed that a 90 ppm ozone treatment for 120 min yielded optimal effectiveness for disinfecting larger volume supplies. Furthermore, a 4000 ppm ozone concentration achieved SARS-CoV-2 RNA elimination over smaller surfaces in 10 min or less, as assessed by RT-qPCR [80].

Surface disinfection plays a pivotal role in combating COVID-19. In response to the health emergency, studies evaluating the efficacy of ozone in deactivating viruses on surfaces or fomites have emerged. Personal protective equipment (PPE) serves as a crucial physical barrier, safeguarding medical personnel. In cases of resource scarcity, reusing PPE underscores the necessity of effective SARS-CoV-2 inactivation. Ozone treatment demonstrated remarkable efficacy in inactivating SARS-CoV-2-contaminated PPE. The capacity of ozone to neutralize SARS-CoV-2 on contaminated protective garments and masks was assessed across various exposure durations, concentrations, and humidity levels. Although the authors confirmed the presence of viral genetic material using RT-PCR, quantification of inactivation remained absent. At ozone concentrations ≥ 2000 ppm, disinfection of SARS-CoV-2 on the surface of contaminated PPE was achieved in under 10 min. Notably, a concentration of 10,000 ppm facilitated disinfection within 30 s. The effects at lower ozone concentrations (4–12 ppm) were heavily contingent upon relative humidity conditions. Nonetheless, the authors concluded that ozonation presented a viable avenue for PPE disinfection. Notably, CT values (where CT represents ozone concentration multiplied by exposure time) of 9.8 mg/L/min were found to render the viral genetic material undetectable (under ≤ 99% relative humidity conditions) [81]. A recent study demonstrated that gaseous ozone at 800 ppm could decontaminate cotton, FFP3 face masks, and glass slides contaminated with SARS-CoV-2 within 10 to 60 min, resulting in a reduction of over 6 logs. This study also highlighted the prevention of ozone-mediated virus inactivation due to surface tension when viral droplets were placed on glass surfaces [82]. DBD plasma (ozone concentration of 120 ppm at a power of 13 W) produced ozone gases capable of eliminating HCoV-229E from the surface of Korean face mask-94 (KF94) under varying exposure times ranging from 10 to 300 s. A 10 s exposure at 120 ppm ozone concentration reduced the viral RNA load by 4 log orders [83]. Another study investigated the inactivation of SARS-CoV-2 and its surrogate, Human Coronavirus OC43 (HCoV-OC43), on representative porous (KN95 mask material) and nonporous surfaces (aluminum and polycarbonate) using a Compact Portable Plasma Reactor (CPPR). Iterative experiments were conducted on inoculated material samples across three operating volumes, and the results showed that minimum CPPR exposure times of 5 to 15 min achieved a 4–5 log reduction in SARS-CoV-2 and its surrogate on the tested materials [84].

Research highlights ozone’s effectiveness in disinfecting surfaces, but it is also important to recognize how different surface materials affect the inactivation of viruses. Specifically, rigid and inert surfaces, such as stainless steel, glass, and plastic, demonstrated similar rates of virus inactivation when treated with ozone. Conversely, porous materials (e.g., floors) or copper surfaces showed the ability to inactivate the virus even in the absence of ozone. Remarkably, copper possesses antimicrobial properties and can deactivate SARS-CoV-2 in minutes [85]. The study by Yano et al. [86] was the pioneering endeavor to demonstrate a 3.3 log_10_ reduction in SARS-CoV-2 on stainless steel plates, achieved through exposure to 6 ppm ozone gas for 55 min at a relative humidity of 60–80%. Subsequently, another study demonstrated the effectiveness of ozonated water (0.2–0.8 ppm) against SARS-CoV-2, resulting in a 2 log_10_ reduction in virus infectivity after just 1 min of exposure [38]. A thorough investigation was conducted to assess the impact of ozone on the viability of SARS-CoV-2 across eight different surfaces, including stainless steel, both painted and unpainted aluminum, acrylic, glass, plastic, FFP2 masks, and surgical gowns. This study took place inside a sealed acrylic box to ensure controlled conditions. While temperature, relative humidity, and ozone emission remained constant, both ozone concentration (ranging from 0.5 to 2 ppm) and exposure time (40 and 60 min) [87] were varied.

To reduce the risk of fomite-mediated transmission of SARS-CoV-2, various surface disinfection and sanitization techniques have been proposed. A study assessing the efficacy and feasibility of gaseous ozone disinfection in a public bus setting used murine hepatitis virus (a surrogate for betacoronaviruses) and *Staphylococcus aureus* as test organisms. The findings indicated that an optimal gaseous ozone regimen achieved a 3.65 log reduction in murine hepatitis virus and a 4.73 log decrease in S. *aureus*. Additionally, decontamination efficacy was influenced by exposure duration and relative humidity within the application space [88]. Frizziero et al. introduced a new sanitization device specifically designed for helmets used in bike-sharing services. This innovative system uses ozone to effectively remove dust and bacteria from the helmet’s surface, addressing rising concerns about hygiene and safety in shared transportation [89].

## 6. Inactivation of SARS-CoV-2 by Ozonated Water and Influencing Factors

A substantial reduction in SARS-CoV-2 titer was observed across the three tested concentrations after a 40 min fumigation period on all surfaces. Notably, the disinfection efficiency did not exhibit a direct proportionality to the concentration used [81]. Westover et al. [90] further substantiated these findings, noting a significant degradation of synthetic SARS-CoV-2 RNA commonly encountered materials: glass, plastic, gauze, wood, fleece, and wool. Ozone exposure at 0.2 ppm over 2 h effectively disinfected wool samples by 99.99%, followed by diminishing efficacy for gauze (96.8%), wood (93.3%), glass (90%), and plastic (82.2%). Similarly, exposing these materials to 4 ppm ozone for 30 min achieved a 90% reduction in viral titers [28].

In addition to the previously discussed factors, the composition of the media plays a crucial role in inactivating viruses. Ozonated water has proven to be an effective alternative for environmental disinfection. Remarkably, just one minute of exposure can achieve a reduction of 2.0 to 5.0 log_10_ in SARS-CoV-2 titers [91]. Tizaoui et al. [92] demonstrated the ability of both aqueous and gaseous ozone to inactivate SARS-CoV-2. The virus was suspended in liquid and also dried on different surfaces, including glass, polystyrene (a type of plastic), copper, stainless steel, ambulance seats, and floor samples. The study revealed a significant difference in ozone flux between liquid and dry surfaces. This differential suggested that rehydrating a dry viral medium amplifies its exposure to ozone, ultimately leading to inactivation [92]. Ozonated water itself demonstrated robust virucidal activity against SARS-CoV-2 [92]. In a study by Hu et al. [93], a mere 1 min exposure to ozonated water (at concentrations of 36 mg/L and 18 mg/L) resulted in complete inactivation of environmental SARS-CoV-2 contamination, with no detection of viral genomic RNA or viral plaques. However, further investigations are warranted to assess the effectiveness of ozone-based water disinfection under real-world conditions [85].

Additionally, the study highlighted the enhancement of microbicidal disinfection efficiency with ozonated water, regardless of the type of disinfectant, which contributed to reduced disinfectant concentrations and mitigated microbial resistance [93]. A study evaluating the effectiveness of different disinfection methods—including conventional chemical methods, ozonated water, and ultraviolet irradiation—on three SARS-CoV-2 variants in hospital settings found that ozonated water at 7 ppm (with a virus-to-test solution ratio of 1:9) did not exhibit clinically significant antiviral activity. The reduction in viral activity was less than 1 log, which is below the virucidal efficacy seen with conventional chemical disinfectants [94]. However, a previous study emphasized that the ratio of virus to test solution is a crucial factor affecting the antiviral effectiveness of ozonated water [95]. To evaluate the impact of organic factors in saliva on the efficacy of ozonated water against SARS-CoV-2, researchers exposed ozonated water to salivary components and measured residual ozone concentrations. Ozonated water inactivated SARS-CoV-2 within 30 s, but its efficacy decreased as protein concentration increased. Higher ozone concentrations (1.5 mg/L) were more effective than lower concentrations (0.5 mg/L). This reduction occurred despite identical initial ozone concentrations and contact times, likely due to ozone degradation by the proteins [96]. Supplementary studies elucidating ozone’s virucidal activity against SARS-CoV-2 are briefly summarized in Table 2.

## 7. Conclusions

In response to the global imperative for enhanced decontamination methods, this comprehensive review underscores the considerable potential of ozone as an efficient and environmentally friendly solution against SARS-CoV-2. Gaseous ozone has proven to be an effective option for inactivating viruses on various surfaces and materials. However, achieving the best treatment results requires careful consideration of its concentration and volume. Similarly, aqueous ozone serves as a viable and sustainable alternative to traditional chemical disinfectants. Together, these findings highlight the potential of ozone as a valuable tool in reducing the transmission of COVID-19. Based on the available body of evidence, our recommendation involves the use of gaseous ozone at concentrations exceeding 4000 ppm, sustained for a minimum of 10 min, to achieve substantial inactivation of SARS-CoV-2 on surfaces and materials.

Aqueous ozone stands out as a highly effective and environmentally friendly disinfectant for liquid media, providing a strong safety profile. Ensuring precise ozone generation and exposure parameters is crucial to eliminating any potential health risks. While the reviewed studies present valuable insights, they also have limitations, including modest sample sizes and variations in experimental conditions. Therefore, continued research is vital to further confirm and enhance the efficacy of ozone in real-world applications against SARS-CoV-2.

## Figures and Tables

**Table 1:** Factors influencing microbial inactivation efficiency during gaseous and aqueous ozonization of different materials.

Material/Substrate	Ambient Conditions	Operational
Temperature	Material shape and porosity	Ozone concentration
pH	Contact angle	Ozonation duration
Conductivity	Contamination method	Ozone generation method
Relative humidity	Level of microbial contamination	Penetrability
Pressure	Surface chemical composition	Radical generation (OH^•^)
Dissolved concentration organic matter		Concentration homogenization

**Table 2:** Ozone virucidal activity against SARS-CoV-2 on different surfaces and other liquid media.

Ozonation Phase	Ozone-Based Device	Ozone Concentration	Exposure Time	Virus Type	Material/Substrate	Summary of Findings	Ref.
Aqueous	Industrial ozone generator (Grenof, Brisbane, Australia).	0.375 ppm, 0.75 ppm, 1 ppm	5 min	SARS-CoV-2, QLD02 (GISAID accession EPI_ISL_407,896) and QLD935 (GISAID accession EPI_ISL_436,097)	Cell line and virus isolates	0.75 ppm aqueous ozone is highly effective in inactivating the virus after 5 min of incubation, with 0.375 ppm achieving 82–91.5% inactivation.	[27]
Aqueous	DOCOL^®^	0.2–0.8 ppm	1 min	SARS-Cov-2 in Vero CCL81 lineage	Virus solution	A reduction in virus infectivity was observed after a 0.6 ppm ozone treatment for 1 min, corresponding to a 2 log_10_ reduction.	[38]
Aqueous	ND	4.5, 9, 18, 36 ppm	1, 5, 10 min	SARS-CoV-2 in Vero E6 cell line	Viral solution	36 ppm aqueous ozonation fully inactivated the virus stock in <1 min.	[92]
Aqueous	Electrolytic ozone water-generating device (Handlex, ONR-1, Nikkiso/Nikkamicron Co., Saitama/Tokyo, Japan)	4, 7, 10 ppm	1, 5–20 s	SARS-CoV-2/Hu/D.P./Kng/19-027, LC528233	Viral stock solution	Virus titer reduction rates after 5 s treatment with ozone concentrations of 1, 4, 7, and 10 ppm were 81.4%, 93.2%, 96.6%, and 96.6%, respectively.	[39]
Aqueous	Ozonated water	7 ppm O3 solution was mixed with the virus (9:1 volume ratio)	3 min and 10 min	Three SARS-CoV-2 variants of concern (WHO label alpha, beta, gamma; Pango lineage B.1.1.7, B.1.351, P.1) in Vero E6 cells (ATCC-CRL1586)	Viral solution	Reduction in the viral activity of <1 log	[94]
Aqueous	Ozone water-generating device (Panasonic, Tokyo, Japan)	1.5 mg/L	5 min	SARS-CoV-2 in Vero E6 cell line	SARS-CoV-2 in saliva	Ozonated water appeared to inactivate SARS-CoV-2 within 30 s. The amount of inactivated SARS-CoV-2 decreased as the protein concentration increased. Virus inactivation was stronger by 1.5 mg/L ozonated water than by 0.5 mg/L ozonated water.	[96]
Gaseous and Aqueous	STERISAFE Pro version 1.0, STERISAFE ApS, Ole Maaløe’s vej 5, DK-2200 Copenhagen, Denmark	0.5–20 g·min/m^3^	3, 5, 20 min	Bacteriophage F6 and BCoV	Stainless steel, copper, plastic, glass, coupons of ambulance seats, and flooring	15 g/min/m^3^ ozone and 81% R.H., a reduction of up to 2 logs was observed on stainless steel and glass. A constant inactivation rate for the liquid was 7 × 10^5^ M^−1^ s^−1^.	[92]
Gaseous/Aqueous	Sanozone™ O3 generator (Sanozone, Regina, SK, Canada)	4.5 and 9 ppm	10–90 min	Recombinant mammalian cell product, bacteriophage MS2, and SARS-CoV-2	Polyester, stainless steel, plastic, and paper. Water treatment was also performed	After 4.5 and 9 ppm ozone exposure for 60–90 min, no virus RNA was recovered from plastic surfaces	[97]
Gaseous/Aqueous	Ozone gas and L CLEAN Minnie (Tamura TECO Co., Ltd., Higashiosaka City, Japan)	0.05–2 ppm	10 s–20 h	SARS-Cov-2 in VeroE6/TMPRSS2 cells	Stainless steel for gaseous exposure and viral solutions.	0.05 and 0.1 ppm ozone gas treatment decreased virus infectivity by ~95% in 10 and 20 h, depending on R.H. 1 and 2 mg/L ozonated water treatment reduced virus infectivity by about 2 and 3 logs in 10 s	[98]
Gaseous and Aqueous	Ozone-gas generator (MXAP-AE270, Maxell Ltd., Tokyo, Japan) and Ozonated water generator (MXZW-WM100J, Maxell Ltd., ejector type	0.05 ppmv and 0.2 mg/L	12–24 h and 30 s and 60 s	SARS-CoV-2; 2019-nCoV JPN/TY/WK-521 strain	Viral solution	Inactivation in the gas phase requires 10^14^–10^15^ ozone molecules per virus virion, while the inactivation in the aqueous phase requires 5 × 10^10^ to 5 × 10^11^ ozone molecules	[99]
Gaseous	ICON3 device	0–5.5 ppm	0–45 min	SARS-CoV-2 in Vero E6 cells (kidney epithelial cells from African green monkey, ATCC CRL-1586)	Virus suspensions in well plates	3.18 ppm of ozone inactivated > 99% of the virus within 20 min.	[78]
Gaseous	Medical ozone generator (Ozonobaric P^®^, Sedecal, Madrid, Spain)	19, 33, 70, and 90 ppm 2000, 4000, 10,000 ppm	30, 60, 90, and 120 min 5, 10 min	SARS-CoV-2 strain 2019-nCoV/USA-WA1/2020	Office supplies (personal computer monitors, keyboards, and mice) and clinical equipment (continuous positive airway pressure tubes and PPE)	4000 ppm ozone eliminated viral RNA within 10 min	[80]
Gaseous	Medical ozone generators (Ozonobaric P^®^, Sedecal, Madrid, Spain)	4–12 ppm 500–40,000 ppm	0.5, 1, 5, 10 min	SARS-CoV-2 strain 2019-nCoV/USA-WA1/2020	PPE gowns and facemasks	No viral amplification was detected after ozone exposure (2000 ppm or higher). At lower concentrations (4–12 ppm), inactivation depended on R.H.	[81]
Gaseous	Plasma generator (CeraPlas™ element, Relyon Plasma GmbH, Regensburg, Germany)	800 ppm	10–60 min	SARS-CoV-2 virus isolate (Human 2019-nCoV Isolate ex China Strain: BavPat1/2020)	Cotton facemasks, FFP3 facemasks, glass slides	800 ppm ozone exposure within 10–60 min demonstrated >6 log reduction	[82]
Gaseous	DBD plasma	120 ppm with power 13 W	10–300 s	HCoV-229E	Face mask-94 (KF94)	Reduce the viral RNA load to 4 log orders with an ozone concentration of 120 ppm, exposed for 10 s	[83]
Gaseous	Device generating ozone gas (TM-04OZ; Tamura TECO Ltd., Higashiosaka City, Osaka, Japan)	1, 6 ppm	55, 60 min	SARS-CoV-2 (JPN/TY/WK-521) strain in VeroE6/TMPRSS2 cells (JCRB1819)	Stainless steel	A 6 ppm ozone gas exposure for 55 min yielded a 3 log_10_ reduction (PFU/mL) in viral load.	[38]
Gaseous	ND	0.5, 1, 2 ppm	40, 60 min	SARS-CoV-2	Painted aluminum, non-painted aluminum, FFP2 masks, glass, plastic, surgical gown, Plexiglas, stainless steel	In 60 min, ~85–90% viability reduction on all surfaces at all concentrations	[88]
Gaseous	Sani Suport Supreme	20 ppm	30 min, 1 h, 2 h, 3 h, 4 h	Twist synthetic SARS-CoV-2 RNA.	Blankets, remotes, catheters, and syringes	At 240 min, ~99% capsid RNA was degraded	[28]
Gaseous	Ozonext Defender 10 (Cea S.p.A., Lecco, Italy)	0.2 and 4 ppm	30–120 min	hCoV-19/Italy/UniSR1/2020 (GISAID accession ID: EPI_ISL_413489) in Vero E6 cells	Wood, gauze, fleece, glass, and plastic	4 ppm ozone exposure within 30 min yielded a 90% reduction in viral titers for all materials	[91]
Gaseous	Bio3gen apparatus (Finlinea s.p.a., Gazzaniga, Bergamo, Italy)	400 mg/h 3.6 L/min	4 min	SARS-CoV-2	Swabs	The real-time detection kit tested negative	[100]
Gaseous	Air sanitizer “Zefero” (Cf7 S.r.l., S. Giovanni La Punta, Catania, Italy)	5 g/h in a chamber of 0.033 m^3^	15, 30 min	Betacoronavirus OC43 and SARS-CoV-2	Aerosol sampling	After 30 min of ozone exposure, the virus was fully eliminated.	[101]
Gaseous	Ozonization system (model GX, Ozotek^®^, Taranto, Italy)	2.64 g/h	2 h 30 min	SARS-CoV-2 isolation in Vero E6 cell line (African green monkey kidney cells)	Supermarket (scales, refrigerator handles, shopping trolley handles, cash register keyboards, and P.O.S. keyboards)	There is no statistically significant difference between supermarkets with and without an ozonation system.	[102]
Gaseous	Ozone generator (Ozonobaric P1, Sedecal, Madrid, Spain)	2000, 4000, and 10,000 ppm	1, 2, 5, and 10 min	SARS-CoV-2 in VERO E6 cells (ATCC CRL-1586)	Face masks, viral solution	The best-evaluated option in this study was 4000 ppm (8 g/m^3^) for 2 min.	[103]
Gaseous	Ozone gas generator (Mitsubishi Heavy Industries, Ltd., Tokyo, Japan).	0.1, 0.3, and 1.0 ppm	0 min, 100 s, 5 min, and 10 min	SARS-CoV-2 strain 2019-nCoV/Japan/TY/WK-521/2020 in VeroE6/TEMPRESS2 cells (JCRB1819)	A mixed sample of SARS-CoV-2 and influenza A virus in diluted saliva	The concentrations of ozone in the air reached a 2 log reduction in SARS-CoV-2 infectivity titer within 10 min at 1.0 ppm.	[79]
Gaseous	Compact Portable Plasma Reactor (CPPR)	1 ppm	5–15 min	SARS-CoV-2 strain UF-1 and HCoV-OC43 strain JAL-1 in Vero E6 cells	SARS-CoV-2 and HCoV-OC43 on representative porous (KN95 mask material) and nonporous materials (aluminum and polycarbonate)	Minimum CPPR exposure times of 5–15 min resulted in a 4–5 log reduction in SARS-CoV-2 and its surrogate on representative material samples.	[84]
Gaseous	Virestorm V5 ozone generator	Between 13 and 18 ppm	55, 85, and 210 min	Murine hepatitis virus (MHV-a related betacoronavirus surrogate) and *Staphylococcus aureus*	Carriers-Stainless steel disks	3.65 log reduction in murine hepatitis virus and a 4.73 log decrease in *S. aureus*	[89]
Glycerol	Ozonizer (Mediplus Co., Tokyo, Japan)	20, 200, 500, and 1000 ppm	20 s, 1 h, and 24 h	SARS-CoV-2 (JPN/TY/WK-521 strain) in VeroE6/TMPRSS2 cells (cell number: JCRB1819)	Viral solution	500 ppm ozonated glycerol at 20% of F.B.S. concentration for 1 h was ≥99.91% of virus inactivation	[95]

ND: Not described.

## References

[1] Mofijur M., Fattah I.M.R., Alam M.A., Islam A.B.M.S., Ong H.C., Rahman S.M.A., Najafi G., Ahmed S.F., Uddin M.A., Mahlia T.M.I. (2021). Impact of COVID-19 on the social, economic, environmental, and energy domains: Lessons learned from a global pandemic. Sustain. Prod. Consum..

[2] Caggiano G., Triggiano F., Apollonio F., Diella G., Lopuzzo M., D’Ambrosio M., Fasano F., Stefanizzi P., Sorrenti G.T., Magarelli P. (2021). SARS-CoV-2 RNA and supermarket surfaces: A real or presumed threat?. Int. J. Environ. Res. Public Health.

[3] Petrillo S., Carrà G., Bottino P., Zanotto E., De Santis M.C., Margaria J.P., Giorgio A., Mandili G., Martini M., Cavallo R. (2020). A novel multiplex qRT-PCR assay to detect SARS-CoV-2 infection: High sensitivity and increased testing capacity. Microorganisms.

[4] Chia P.Y., Coleman K.K., Tan Y.K., Ong S.W.X., Gum M., Lau S.K., Lim X.F., Lim A.S., Sutjipto S., Lee P.H. (2020). Singapore 2019 Novel Coronavirus Outbreak Research Team. Detection of air and surface contamination by SARS-CoV-2 in hospital rooms of infected patients. Nat. Commun..

[5] Mondelli M.U., Colaneri M., Seminari E.M., Baldanti F., Bruno R. (2021). Low risk of SARS-CoV-2 transmission by fomites in real-life conditions. Lancet Infect. Dis..

[6] Harvey A.P., Fuhrmeister E.R., Cantrell M.E., Pitol A.K., Swarthout J.M., Powers J.E., Nadimpalli M.L., Julian T.R., Pickering A.J. (2021). Longitudinal monitoring of SARS-CoV-2 RNA on high-touch surfaces in a community setting. Environ. Sci. Technol. Lett..

[7] Liu Y., Li T., Deng Y., Liu S., Zhang D., Li H., Wang X., Jia L., Han J., Bei Z. (2021). Stability of SARS-CoV-2 on environmental surfaces and in human excreta. J. Hosp. Infect..

[8] Van Doremalen N., Bushmaker T., Morris D.H., Holbrook M.G., Gamble A., Williamson B.N., Tamin A., Harcourt J.L., Thornburg N.J., Gerber S.I. (2020). Aerosol and surface stability of SARS-CoV-2 as compared with SARS-CoV-1. N. Engl. J. Med..

[9] Jayaweera M., Perera H., Gunawardana B., Manatunge J. (2020). Transmission of COVID-19 virus by droplets and aerosols: A critical review on the unresolved dichotomy. Environ. Res..

[10] Aboubakr H.A., Sharafeldin T.A., Goyal S.M. (2020). Stability of SARS-CoV and other coronaviruses in the environment and on common touch surfaces and the influence of climatic conditions: A review. Transbound. Emerg. Dis..

[11] Irie M.S., Dietrich L., Souza G.L., Soares P.B.F., Moura C.C.G., Silva G.R.D., Paranhos L.R. (2022). Ozone disinfection for viruses with applications in healthcare environments: A scoping review. Braz. Oral. Res..

[12] Gola M., Caggiano G., De Giglio O., Napoli C., Diella G., Carlucci M., Carpagnano L.F., D’Alessandro D., Joppolo C.M., Capolongo S. (2021). SARS-CoV-2 indoor contamination: Considerations on anti-COVID-19 management of ventilation systems and finishing materials in healthcare facilities. Ann. Ig..

[13] Rangel K., De-Simone S.G. (2023). *Acinetobacter baumannii* during COVID-19: What is the real pandemic?. Pathogens.

[14] Tregoning J.S., Wang Z., Sridhar S., Shattock R.J., DeRosa F. (2025). Immunology of RNA-based vaccines: The critical interplay between inflammation and expression. Mol. Ther..

[15] Moghadas S.M., Vilches T.N., Zhang K., Wells C.R., Shoukat A., Singer B.H., Meyers L.A., Neuzil K.M., Langley J.M., Fitzpatrick M.C. (2021). The impact of vaccination on COVID-19 outbreaks in the United States. Clin. Infect. Dis..

[16] Tenforde M.W., Olson S.M., Self W.H., Talbot H.K., Lindsell C.J., Steingrub J.S., Shapiro N.I., Ginde A.A., Douin D.J., Prekker M.E. (2021). Effectiveness of Pfizer-BioNTech and Moderna vaccines against COVID-19 among hospitalized adults aged ≥65 years—United States January-March 2021. MMWR Morb. Mortal. Wkly. Rep..

[17] Leung K., Shum M.H., Leung G.M., Lam T.T., Wu J.T. (2021). Early transmissibility assessment of the N501Y mutant strains of SARS-CoV-2 in the United Kingdom: October to November 2020. Euro Surveill..

[18] Makoni M. (2021). South Africa responds to new SARS-CoV-2 variant. Lancet.

[19] Dewey H.M., Jones J.M., Keating M.R., Budhathoki-Uprety J. (2022). Increased use of disinfectants during the COVID-19 pandemic and its potential impacts on health and safety. ACS Chem. Health Saf..

[20] Manjunath S.N., Sakar M., Katapadi M., Geetha Balakrishna R. (2021). Recent case studies on the use of ozone to combat coronavirus: Problems and perspectives. Environ. Technol. Innov..

[21] Rangel K., Cabral F.O., Lechuga G.C., Carvalho J.P.R.S., Villas-Bôas M.H.S., Midlej V., De-Simone S.G. (2022). Detrimental effect of ozone on pathogenic bacteria. Microorganisms.

[22] Rangel K., Cabral F.O., Lechuga G.C., Carvalho J.P.R.S., Villas-Bôas M.H.S., Midlej V., De-Simone S.G. (2022). Potent activity of high concentrations of chemical ozone against antibiotic-resistant bacteria. Molecules.

[23] Liu J., Yue P., Huang L., Zhao M., Kang X., Liu X. (2020). Styrene removal with an acidic biofilter with four packing materials: Performance and fungal bioaerosol emissions. Environ. Res..

[24] Zanardi I., Borrelli E., Valacchi G., Travagli V., Bocci V. (2015). Ozone: A multifaceted molecule with unexpected therapeutic activity. Curr. Med. Chem..

[25] Shen J., Gao Z. (2018). Ozone removal on building material surface: A literature review. Build. Environ..

[26] (2020). Istituto Superiore di Sanità, Focus on the Professional Use of Ozone Also in Reference to COVID-19 Working Group ISS-INAIL. https://www.iss.it/en/pubblicazioni-focus/-/asset_publisher/GIDBUf2rmBr2/content/id/5524886.

[27] Albert S., Amarilla A.A., Trollope B., Sng J.D.J., Setoh Y.X., Deering N., Modhiran N., Weng S.H., Melo M.C., Hutley N. (2021). Assessing the potential of unmanned aerial vehicle spraying of aqueous ozone as an outdoor disinfectant for SARS-CoV-2. Environ. Res..

[28] Criscuolo E., Diotti R.A., Ferrarese R., Alippi C., Viscardi G., Signorelli C., Mancini N., Clementi M., Clementi N. (2021). Fast inactivation of SARS-CoV-2 by UV-C and ozone exposure on different materials. Emerg. Microbes Infect..

[29] Martínez-Sánchez G., Schwartz A., Donna V.D. (2020). Potential cytoprotective activity of ozone therapy in SARS-CoV-2/COVID-19. Antioxidants.

[30] Yousefi B., Banihashemian S.Z., Feyzabadi Z.K., Hasanpour S., Kokhaei P., Abdolshahi A., Emadi A., Eslami M. (2022). Potential therapeutic effect of oxygen-ozone in controlling of COVID-19 disease. Med. Gas Res..

[31] Shang W., Wang Y., Wang G., Han D. (2023). Benefits of ozone on mortality in patients with COVID-19: A systematic review and meta-analysis. Complement. Ther. Med..

[32] Jafari-Oori M., Vahedian-Azimi A., Ghorbanzadeh K., Sepahvand E., Dehi M., Ebadi A., Izadi M. (2022). Efficacy of ozone adjuvant therapy in COVID-19 patients: A meta-analysis study. Front. Med..

[33] Budi D.S., Rofananda I.F., Pratama N.R., Sutanto H., Hariftyani A.S., Desita S.R., Rahmasari A.Z., Asmarawati T.P., Waskito L.A., Wungu C.D.K. (2022). Ozone as an adjuvant therapy for COVID-19: A systematic review and meta-analysis. Int. Immunopharmacol..

[34] Cenci A., Macchia I., La Sorsa V., Sbarigia C., Di Donna V., Pietraforte D. (2022). Mechanisms of action of ozone therapy in emerging viral diseases: Immunomodulatory effects and therapeutic advantages with reference to SARS-CoV-2. Front. Microbiol..

[35] Smith N.L., Wilson A.L., Gandhi J., Vatsia S., Khan S.A. (2017). Ozone therapy: An overview of pharmacodynamics current research and clinical utility. Med. Gas. Res..

[36] Bocci V., Valacchi G., Corradeschi F., Fanetti G. (1998). Studies on the biological effects of ozone: 8. Effects on the total antioxidant status and interleukin-8 production. Mediat. Inflamm..

[37] Ranaldi G.T., Villani E.R., Franza L. (2020). Rationale for ozone-therapy as adjuvant therapy in COVID-19: A narrative review. Med. Gas. Res..

[38] Martins R.B., Castro I.A., Pontelli M., Souza J.P. (2021). SARS-CoV-2 inactivation by ozonated water: A preliminary alternative for environmental disinfection. Ozone Sci. Eng..

[39] Inagaki H., Akatsuki S., Sudaryatma P.E., Sugiyamaa H., Okabayash Y., Fujimoto S. (2021). Rapid inactivation of SARS-CoV-2 with ozonated water. Ozone Sci. Eng..

[40] El Meligy O.A., Elemam N.M., Talaat I.M. (2023). Ozone therapy in medicine and dentistry: A review of the literature. Dent. J..

[41] Hudson J.B., Sharma M., Vimalanathan S. (2009). Development of a practical method for using ozone gas as a virus decontaminating agent. Ozone Sci. Eng..

[42] Morrison C., Atkinson A., Zamyadi A., Kibuye F., McKie M., Hogard S., Mollica P., Jasim S., Wert E.C. (2021). Critical review and research needs of ozone applications related to virus inactivation: Potential implications for SARS-CoV-2. Ozone Sci. Eng..

[43] Hoigne L., Baderm H. (1979). Ozonation of water: Selectivity and rate of oxidation of solutes. Ozone Sci. Eng..

[44] Viebahn-Haensler R., León Fernández O.S. (2021). Ozone in medicine. The low-dose ozone concept and its basic biochemical mechanisms of action in chronic inflammatory diseases. Int. J. Mol. Sci..

[45] Kumar G.D., Williams R.C., Sumnerm S.S., Eifertm J.D. (2016). Effect of ozone and ultraviolet light on Listeria monocytogenes populations in fresh and spent chill brines. Food Cont..

[46] Guzel-Seydim Z.B., Greene A.K., Seydim A.C. (2004). Use of ozone in the food industry. LWT-Food Sci. Technol..

[47] Joseph C.G., Farm Y.Y., Taufiq-Yap Y.H., Pang C.K., Nga J.L.H., Puma G.L. (2021). Ozonation treatment processes for the remediation of detergent wastewater: A comprehensive review. J. Environ. Chem. Eng..

[48] Mousazadeh M., Kabdaşlı I., Khademi S., Sandoval M.A., Moussavi S.P., Malekdar F., Gilhotra V., Hashemi M., Dehghani M.H. (2022). A critical review on the existing wastewater treatment methods in the COVID-19 era: What is the potential of advanced oxidation processes in combatting viral especially SARS-CoV-2?. J. Water Process Eng..

[49] Pawrat J., Terebun P., Kwiatkowski M., Tarabová B., Kovaľová Z., Kučerová K., Machala Z., Janda M., Hensel K. (2019). Evaluation of oxidative species in gaseous and liquid phase generated by mini-gliding arc discharge. Plasma Chem. Plasma Process.

[50] Dobslaw D., Schulz A., Helbich S., Dobslaw C. (2017). VOC removal and odor abatement by a low-cost plasma enhanced bio trickling filter process. J. Environ. Chem. Eng..

[51] Dobslaw D., Ortlinghaus O., Dobslaw C. (2018). A combined process of non-thermal plasma and a low-cost mineral adsorber for V.O.C. removal and odor abatement in emissions of organic waste treatment plants. J. Environ. Chem. Eng..

[52] Park C.H., Byeon J.H., Yoon K.Y., Park J., Hwang J. (2011). Simultaneous removal of odors airborne particles and bioaerosols in a municipal composting facility by dielectric barrier discharge. Sep. Purif. Technol..

[53] Claus H. (2021). Ozone generation by ultraviolet lamps. Photochem. Photobiol..

[54] Boelter K.J., Davidson J.H. (2007). Ozone generation by indoor electrostatic air cleaners. Aerosol Sci. Technol..

[55] Caniani D., Caivano M., Mazzone G., Mais S., Mancini I.M. (2021). Effect of site-specific conditions and operating parameters on the removal efficiency of petroleum-originating pollutants using ozonation. Sci. Total Environ..

[56] Egorova G.V., Voblikova V.A., Sabitova L.V., Tkachenko I.S. (2015). Ozone solubility in water. Mosc. Univ. Chem. Bull..

[57] Bayarri B., Cruz-Alcalde A., Lopez-Vinent N., Micó M.M., Sans C. (2021). Can ozone inactivate SARS-CoV-2? A review of mechanisms and performance on viruses. J. Hazard. Mater..

[58] Tizaoui C. (2020). Ozone: A potential oxidant for COVID-19 Virus (SARS-CoV-2). Ozone Sci. Eng..

[59] Skowron K., Wałecka-Zacharska E., Grudlewska K., Białucha A., Wiktorczyk N., Bartkowska A., Kowalska M., Kruszewski S., Gospodarek-Komkowska E. (2019). Biocidal effectiveness of selected disinfectant solutions based on water and ozonated water against *Listeria monocytogenes* strains. Microorganisms.

[60] Erickson M.C., Ortega Y.R. (2006). Inactivation of protozoan parasites in food, water, and environmental systems. J. Food Prot..

[61] Epelle E.I., Macfarlane A., Cusack M., Burns A., Okolie J.A., Mackay W., Rateb M., Yaseen M. (2023). Ozone application in different industries: A review of recent developments. Chem. Eng. J..

[62] Cai Y., Zhao Y., Yadav A.K., Ji B., Kang P., Wei T. (2023). Ozone based inactivation and disinfection in the pandemic time and beyond: Taking forward what has been learned and best practice. Sci. Total Environ..

[63] Dubuis M.E., Dumont-Leblond N., Lalibert’e C., Veillette M., Turgeon N., Jean J., Duchaine C. (2015). Ozone efficacy for the control of airborne viruses: Bacteriophage and norovirus models. PLoS ONE.

[64] Cristiano L. (2020). Could ozone be an effective disinfection measure against the novel Coronavirus (SARS-CoV-2)?. J. Prev. Med. Hyg..

[65] Kong J., Lu Y., Ren Y., Chen Z., Chen M. (2021). The virus removal in U.V. irradiation ozonation and chlorination. Water Cycle.

[66] Fehr A.R., Perlman S., Maier H.J., Bickerton E., Britton P. (2015). Coronaviruses: An overview of their replication and pathogenesis. Coronaviruses: Methods and Protocols.

[67] Zhou P., Yang X., Wang X., Hu B., Zhang L., Zhang W., Si H.R., Zhu Y., Li B., Huang C.L. (2020). A pneumonia outbreak associated with a new coronavirus of probable bat origin. Nature.

[68] Murray B.K., Ohmine S., Tomer D.P., Jensen K.J., Johnson F.B., Kirsi J.J., Robison R.A., O’Neill K.L. (2008). Virion disruption by ozone-mediated reactive oxygen species. J. Virol. Methods.

[69] Hirneisen K.A., Markland S.M., Kniel K.E. (2011). Ozone inactivation of norovirus surrogates on fresh produce. J. Food Prot..

[70] Wolf C., Von Gunten U., Kohn T. (2018). Kinetics of inactivation of waterborne enteric viruses by ozone. Environ. Sci. Technol..

[71] Li C.S., Wang Y.C. (2003). Surface germicidal effects of ozone for microorganisms. AIHAJ Am. Ind. Hyg. Assoc..

[72] Young S., Torrey J., Bachmann V., Kohn T. (2020). Relationship between inactivation and genome damage of human enteroviruses upon treatment by UV254 free chlorine and ozone. Food Environ. Virol..

[73] Allison K., Hook J., Cardis D., Rice R.G. (2009). Quantification of the bactericidal fungicidal and sporicidal efficacy of the J.L.A. Ltd ozone laundering system. Ozone Sci. Eng..

[74] Wigginton K.R., Kohn T. (2012). Virus disinfection mechanisms: The role of virus composition structure and function. Curr. Opin. Virol..

[75] Uppal T., Khazaieli A., Snijders A.M., Verma S.C. (2021). Inactivation of human coronavirus by FATHHOME’s dry sanitizer device: Rapid and eco-friendly ozone-based disinfection of SARS-CoV-2. Pathogens.

[76] Franke G., Knobling B., Brill F.H., Becker B., Klupp E.M., Belmar Campos C., Pfefferle S., Lütgehetmann M., Knobloch J.K. (2021). An automated room disinfection system using ozone is highly active against surrogates for SARS-CoV-2. J. Hosp. Infect..

[77] Yao M., Zhang L., Ma J., Zhou L. (2020). On airborne transmission and control of SARS-CoV-2. Sci. Total Environ..

[78] De Forni D., Poddesu B., Cugia G., Gallizia G., La Licata M., Lisziewicz J., Chafouleas J., Lori F. (2024). Low ozone concentration and negative ions for rapid SARS-CoV-2 inactivation. J. Biotechnol. Biomed..

[79] Imoto Y., Matsui H., Ueda C., Nakajima E., Hanaki H. (2025). Inactivation effects of hypochlorous acid, chlorine dioxide, and ozone on airborne SARS-CoV-2 and influenza A virus. Food Environ. Virol..

[80] Torres-Mata L.B., García-Pérez O., Rodríguez-Esparragón F., Blanco A., Villar J., Ruiz-Apodaca F., Martín-Barrasa J.L., González-Martín J.M., Serrano-Aguilar P., Piñero J.E. (2022). Ozone eliminates SARS-CoV-2 from difficult-to-clean office supplies and clinical equipment. Int. J. Environ. Res. Public Health.

[81] Clavo B., Cordoba-Lanús E., Rodríguez-Esparragon F., Cazorla-Rivero S.E., García-Pérez O., Piñero J.E., Villar J., Blanco A., Torres-Ascensión C., Martín-Barrasa J.L. (2020). Effects of ozone treatment on personal protective equipment contaminated with SARS-CoV-2. Antioxidants.

[82] Wolfgruber S., Loibner M., Puff M., Melischnig A., Zatloukal K. (2022). SARS-CoV2 neutralizing activity of ozone on porous and nonporous materials. New Biotechnol..

[83] Choi E.H., Uhm H.S., Kaushik N.K. (2021). Plasma bioscience and its application to medicine. AAPPS Bull..

[84] Choudhury B., Lednicky J.A., Loeb J.C., Portugal S., Roy S. (2024). Inactivation of SARS CoV-2 on porous and nonporous surfaces by compact portable plasma reactor. Front. Bioeng. Biotechnol..

[85] Nakano R., Nakano A., Sasahara T., Suzuki Y., Nojima Y., Yano H. (2025). Antiviral effects of copper and copper alloy and the underlying mechanisms in severe acute respiratory syndrome coronavirus 2. J. Hazard. Mater. Adv..

[86] Yano H., Nakano R., Suzuki Y., Nakano A., Kasahara K., Hosoi H. (2020). Inactivation of severe acute respiratory syndrome coronavirus 2 (SARS-CoV-2) by gaseous ozone treatment. J. Hosp. Infect..

[87] Percivalle E., Clerici M., Cassaniti I., Vecchio Nepita E., Marchese P., Olivati D., Catelli C., Berri A., Baldanti F., Marone P. (2021). SARS-CoV-2 viability on different surfaces after gaseous ozone treatment: A preliminary evaluation. J. Hosp. Infect..

[88] Neves E.S., Ng C.T., Pek H.B., Goh V.S.L., Mohamed R., Osman S., Ng Y.K., Kadir S.A., Nazeem M., She A. (2023). Field trial assessing the antimicrobial decontamination efficacy of gaseous ozone in a public bus setting. Sci. Total Environ..

[89] Frizziero L., Donnici G., Venditti G., Freddi M. (2024). Design of an innovative sanitation system for bike-sharing service. Heliyon.

[90] Westover C., Rahmatulloev S., Danko D., Afshin E.E., O’Hara N.B., Ounit R., Bezdan D., Mason C.E. (2022). Ozone treatment for elimination of bacteria and SARS-CoV-2 for medical environments. Genes.

[91] Viana Martins C.P., Xavier C.S.F., Cobrado L. (2022). Disinfection methods against SARS-CoV-2: A systematic review. J. Hosp. Infect..

[92] Tizaoui C., Stanton R., Statkute E., Rubina A., Lester-Card E., Lewis A., Holliman P., Worsley D. (2022). Ozone for SARS-CoV-2 inactivation on surfaces and in liquid cell culture media. J. Hazard. Mater..

[93] Hu X., Chen Z., Su Z., Deng F., Chen X., Yang Q., Li P., Chen Q., Ma J., Guan W. (2021). Ozone water is an effective disinfectant for SARS-CoV-2. Virol. Sin..

[94] Corzo-Leon D.E., Abbood H.M., Colamarinom R.A., Steiner M.F.C., Munro C., Gould I.M., Hijazi K. (2024). Methods for SARS-CoV-2 hospital disinfection, *in vitro* observations. Infect. Prev. Pract..

[95] Takeda Y., Jamsransuren D., Makita Y., Kaneko A., Matsuda S., Ogawa H., Oh H. (2021). Inactivation activities of ozonated water, slightly acidic electrolyzed water and ethanol against SARS-CoV-2. Molecules.

[96] Yasugi M., Gunji K., Inagaki K., Kuroda M., Ii C. (2025). Disinfection effect of ozonated water on SARS-CoV-2 in the presence of salivary proteins. J. Hosp. Infect..

[97] Volkoff S.J., Carlson T.J., Leik K., Smith J.J. (2021). Demonstrated SARS-CoV-2 surface disinfection using ozone. Ozone Sci. Eng..

[98] Murata T., Komoto S., Iwahori S., Sasaki J., Nishitsuji H., Hasebe T., Hoshinaga K., Yuzawa Y. (2021). Reduction of severe acute respiratory syndrome Coronavirus-2 infectivity by admissible concentration of ozone gas and water. Microbiol. Immunol..

[99] Nishiki Y., Imazu T., Nakamuro K., Naitou H., Aoki K.J. (2023). Inactivation mechanism of SARS-CoV-2 by ozone in aqueous and gas phases. J. Microorg. Control.

[100] Sallustio F., Cardinale G., Voccola S., Picerno A., Porcaro P., Gesualdo L. (2021). Ozone eliminates novel coronavirus SARS-CoV-2 in mucosal samples. New Microbes New Infect..

[101] Nicolo M.S., Rizzo M.G., Palermo N., Gugliandolo C., Cuzzocrea S., Guglielmino S.P.P. (2022). Evaluation of betacoronavirus OC43 and SARS-CoV-2 elimination by zefero air sanitizer device in a novel laboratory recirculation system. Pathogens.

[102] Diella G., Caggiano G., Apollonio F., Triggiano F., Stefanizzi P., Fasano F., Pace L., Marcotrigiano V., Sorrenti D.P., Sorrenti G.T. (2023). SARS-CoV-2 RNA viability on high-touch surfaces and evaluation of a continuous-flow ozonation treatment. Ann. Ig..

[103] Córdoba-Lanús E., García-Pérez O., Rodríguez-Esparragón F., Bethencourt-Estrella C.J., Torres-Mata L.B., Blanco A., Villar J., Sanz O., Díaz J.J., Martín-Barrasa J.L. (2022). Ozone treatment effectively eliminates SARS-CoV-2 from infected face masks. PLoS ONE.

